# Lipid nanoparticle siRNA cocktails for the treatment of mantle cell lymphoma

**DOI:** 10.1002/btm2.10088

**Published:** 2018-04-06

**Authors:** Christopher M. Knapp, Jia He, John Lister, Kathryn A. Whitehead

**Affiliations:** ^1^ Dept. of Chemical Engineering; ^2^ Dept. of Biomedical Engineering Carnegie Mellon University, 5000 Forbes Ave. Pittsburgh PA 15213; ^3^ Div. of Hematology and Cellular Therapy Allegheny Health Network Cancer Institute Pittsburgh PA 15224

**Keywords:** cancer therapy, lipid nanoparticles, lipidoid, apoptosis, lymphoma, siRNA

## Abstract

Mantle cell lymphoma is an aggressive and incurable subtype of non‐Hodgkin B cell lymphoma. Patients typically present with advanced disease, and most patients succumb within a decade of diagnosis. There is a clear and urgent need for novel therapeutic approaches that will affect mantle cell lymphoma through a unique mechanism compared to current therapies. This study examined the use of RNA interference (RNAi) therapy to attack mantle cell lymphoma at the mRNA level, silencing genes associated with cancer cell proliferation. We identified a lipid nanoparticle formulated with the lipidoid 306O_13_ that delivered siRNA to JeKo‐1 and MAVER‐1 mantle cell lymphoma cell lines. Three therapeutic gene targets were examined for their effect on lymphoma growth. These included Cyclin D1, which is a cell cycle regulator, as well as Bcl‐2 and Mcl‐1, which prevent apoptosis. Gene knockdown with siRNA doses as low at 10 nM increased lymphoma cell apoptosis without carrier‐mediated toxicity. Silencing of Cyclin D1 induced apoptosis despite a twofold “compensation” upregulation of Cyclin D2. Upon simultaneous silencing of all three genes, nearly 75% of JeKo‐1 cells were apoptosing 3 days post‐transfection. Furthermore, cells proliferated at only 15% of their pretreatment rate. These data suggest that lipid nanoparticles‐formulated, multiplexed siRNA “cocktails” may serve as a beneficial addition to the treatment regimens for mantle cell lymphoma and other aggressive cancers.

## 1 INTRODUCTION

Non‐Hodgkin lymphoma is a type of cancer that originates in secondary lymphoid organs such as the spleen and lymph nodes. In 2016, there were an estimated 73,000 new non‐Hodgkin lymphoma cases and 20,000 deaths in the United States, making it the seventh most deadly cancer in the United States.^1^ The current prognosis for patients not enrolled in a clinical trial is dire, as their life expectancy post‐diagnosis is only 3 years.^2^ A significant hurdle preventing better outcomes is the high dose of chemotherapy required to induce remission. Although there is no gold standard for mantle cell lymphoma therapy, “fit” patients are generally treated with intense chemotherapy regimens such as R‐hyper‐CVAD (rituximab, cyclophosphamide, vincristine, doxorubicin, dexamethasone) alternating with high dose methotrexate and cytarabine.^3^ The remaining patients typically receive R‐CHOP (rituximab, cyclophosphamide, vincristine, prednisone, and doxorubicin).^4^ Several therapeutics with novel mechanisms of action have shown activity in MCL, but without cure. These include bortezomib, ibrutinib, and the recently approved acalabrutinib. Although initial treatment typically results in remission, mantle cell lymphoma is prone to relapse, with progression free survival limited to less than 2 years regardless of treatment approach.^3^, ^5^ Unfortunately, treatment options are limited upon relapse, in part due to the cardiotoxic nature of chemotherapy and limitations on the total dose that can be tolerated in a lifetime.^6^, ^7^, ^8^

Historically, treatment regimens for mantle cell lymphoma and other non‐Hodgkin lymphomas have become more effective with the addition of mechanistically distinct forms of therapy.^9^, ^10^, ^11^, ^12^ Considering that chemotherapy and immunotherapy take effect at the DNA and protein levels, RNA interference (RNAi) therapy may offer a viable means to kill cancer at the mRNA level. When used in combination with current treatments, RNAi may help to induce remission using lower doses of chemotherapy, which would better preserve treatment options upon relapse. Furthermore, an RNAi approach is apropos because mantle cell lymphoma cells overexpress several genes that encourage cell proliferation.^13^, ^14^ For example, Cyclin D1 (CCND1), a protein that facilitates cell cycle progression, is overexpressed in more than 90% of mantle cell lymphoma patients due to a *t*(11;14) (q13;q32) translocation of Cyclin D1 and immunoglobulin heavy chain genes (IgH).^5^ Additionally, the anti‐apoptotic proteins Bcl‐2 and Mcl‐1 are commonly overexpressed in mantle cell lymphoma and may contribute to chemotherapy resistance.^15^, ^16^, ^17^ Although protein inhibitors of Bcl‐2 and Mcl‐1 have shown promise,^18^, ^19^, ^20^, ^21^, ^22^ downregulation at the mRNA level with a viable delivery system has been overlooked.

Unfortunately, B‐cells are notoriously difficult to transfect, and only a limited number of studies have reported on short interfering RNA (siRNA) delivery to lymphoma cells.^23^, ^24^, ^25^, ^26^, ^27^ For example, we have previously described the ability of siRNA‐loaded lipid nanoparticles (LNPs) to silence Mcl‐1 expression and increase the apoptosis rates of mantle cell lymphoma.^28^ Because our LNPs are potent transfection agents,^29^, ^30^, ^31^, ^32^, ^33^ we herein investigate their use for multiplexed gene silencing in mantle cell lymphoma cells. Our data show that the simultaneous silencing of three key genes involved in mantle cell lymphoma growth induces apoptosis in the majority of lymphoma cells. As such, siRNA “cocktails” may hold promise as a mechanistically distinct addition to current mantle cell lymphoma treatment regimens.

## 2 RESULTS

**Figure 1:**
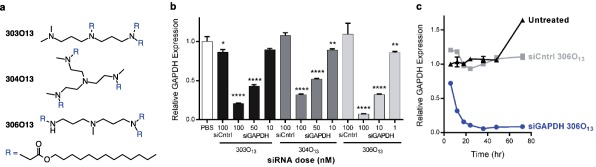
LNPs mediated durable gene silencing in JeKo‐1 cells. (a) Three lipidoid chemistries—303O_13_, 304O_13_, and 306O_13_—were formulated into siRNA‐loaded LNPs. (b) Treatment with each LNP resulted in dose‐dependent silencing of GAPDH in JeKo‐1 mantle cell lymphoma cells 24 hr post‐transfection. PBS and LNPs (siCntrl) at the maximum dose of 100 nM served as negative controls. (c) Following a single dose of 100 nM siGAPDH, 306O_13_ LNPs mediated near‐complete GAPDH knockdown for at least 3 days with maximal silencing achieved by 36 hr (blue circles). In each panel, error bars represent standard deviation (*n* = 3). Statistically significant differences compared to cells given PBS were determined using two‐tailed Welch's *t* tests. *, **, and **** indicate *p* ≤ .05, 0.01, and .0001, respectively

In this study, we sought to identify an RNAi treatment with potential to enhance multi‐pronged mantle cell lymphoma therapy. Mantle cell lymphoma patients have a poor prognosis and patients that do not participate in clinical trials have a life expectancy of only 3 years after initial diagnosis.^2^, ^4^ The genetic mutations present in many mantle cell lymphoma patients make this malignancy an ideal candidate for RNA interference therapy.^25^, ^34^ Although lymphocytes can be particularly challenging to transfect compared to monocytes and adherent cells, we have previously established that lipidoid‐containing LNPs facilitate gene silencing in mantle cell lymphoma cells.^28^ In the present study, we first evaluated the ability of three lipidoids—303O_13_, 304O_13_, and 306O_13_ (Figure 1a)—to durably silence mRNA expression in JeKo‐1 immortalized mantle cell lymphoma cells. Our goal was to identify the most potent lipidoid, as it would be the best candidate for multiplexed gene silencing. For all three lipidoids, formulated nanoparticles were ∼70–100 nm in diameter with PDI <0.15 and siRNA entrapments greater than 75%.

### 2.1 LNPs potently silenced GAPDH in human mantle cell lymphoma cells in vitro

LNPs containing the lipidoids 303O_13_, 304O_13_, and 306O_13_ were examined for their ability to silence the housekeeping gene glyceraldehyde‐3‐phosphate dehydrogenase (GAPDH) in vitro. Following a 24 hr incubation period with cells, each LNP mediated dose‐responsive GAPDH knockdown (Figure 1b). The lipidoid 306O_13_ was the most potent of the three, with an EC_50_ of less than 10 nM siGAPDH. No significant reduction in gene expression was observed for cells treated with control 306O_13_ LNPs, which contained 100 nM siGFP. Additionally, no reduction in cell viability was observed in cells treated with 306O_13_ LNPs (Supporting Information Figure 1). 306O_13_ LNPs also silenced GAPDH (albeit more modestly) in MAVER‐1 cells, a more aggressive human mantle cell lymphoma line, in a dose‐responsive manner (Supporting Information Figure 2). We anticipate that this ability to mediate silencing in lymphoma cells with varying degrees of gene mutation will be important in developing a broadly applicable therapeutic. Because of their higher potency compared to 303O_13_ and 304O_13_ LNPs, we opted to work with 306O_13_ LNPs for the remainder of our studies. A time point study was conducted to examine the duration of silencing in JeKo‐1 cells treated with 306O_13_ LNPs. Maximal gene silencing (94.3 ± 0.3%) was reached by 36 hr following treatment with a single 100 nM dose of siGAPDH 306O_13_ LNPs and was sustained for at least 72 hr (Figure 1c).

### 2.2 LNPs silenced genes commonly overexpressed in mantle cell lymphoma

After establishing that three‐tailed 306O_13_ is the most potent LNP for gene silencing in mantle cell lymphoma cells, we turned our focus to three genes that are upregulated in mantle cell lymphoma patients. These included the cell cycle regulator, Cyclin D1, and the antiapoptotic proteins, B‐cell lymphoma 2 (Bcl‐2), and myeloid cell leukemia 1 (Mcl‐1). Cyclin D1 facilitates cell cycle progression from the G1 to the S phase through complex formation with Cyclin D kinases 4 and 6, which phosphorylate retinoblastoma 1.^35^ Cyclin D1 overexpression occurs in more than 90% of mantle cell lymphoma patients as a consequence of a *t*(11;14) (q13;q32) translocation of Cyclin D1 and the IgH heavy chain.^36^ This overexpression deregulates the cell cycle, leading to rapid progression from the G1 to the S phase and subsequent proliferation. It has been shown that Cyclin D1 downregulation leads to cell cycle arrest, apoptosis, and an increased sensitivity to chemotherapy.^23^, ^24^, ^25^

Bcl‐2 and Mcl‐1 are antiapoptotic proteins that are also commonly overexpressed in mantle cell lymphoma. These proteins inhibit apoptosis by binding and sequestering two pro‐apoptotic proteins, BAX and BAK. BAX and BAK's presence on the mitochondrial membrane is needed for the release of cytochrome c to the cytoplasm allowing for the activation of caspases during apoptosis.^16^, ^37^, ^38^, ^39^, ^40^ Downregulation of Bcl‐2 has been shown to increase apoptosis in mantle cell lymphoma cells treated with immunotoxins in vitro.^41^ Other studies have demonstrated that Mcl‐1 downregulation increases apoptosis in a variety of cancers.^37^, ^42^, ^43^

**Figure 2:**
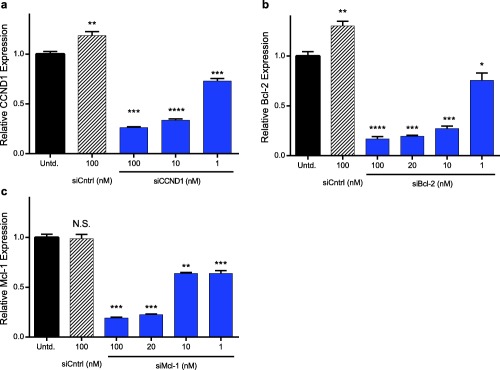
The LNP 306O_13_ potently silenced therapeutic gene targets in JeKo‐1 cells. Silencing of three genes: (a) Cyclin D1 (CCND1), (b) Bcl‐2, and (c) Mcl‐1 in JeKo‐1 cells 24 hr following transfection with 1–100 nM of the relevant siRNA was dose responsive. mRNA expression for each gene was calculated relative to untreated cells. In each panel, error bars represent standard deviation (*n* = 3). Statistically significant differences compared to untreated cells were determined using two‐tailed Welch's t tests. *, **, ***, and **** indicate *p* ≤ .05, 0.01, .001, and .0001, respectively

LNPs facilitated the silencing of each of these genes at the mRNA level in JeKo‐1 cells (Figure 2). Gene knockdown was dose‐responsive, with the highest siRNA dose of 100 nM facilitating 75–80% silencing for each gene. LNPs mediated similar levels of silencing for each gene target, with Mcl‐2 being slightly more difficult to knockdown compared to Cyclin D1 and Bcl‐2. No reduction in gene expression was observed for control LNPs, suggesting that gene silencing was not due to nonspecific delivery vehicle induced cytotoxicity.

**Figure 3:**
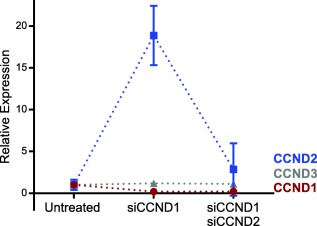
Silencing Cyclin D1 upregulated Cyclin D2 mRNA expression. Twenty‐four hours following transfection of JeKo‐1 cells with 50 nM of anti‐Cyclin D1 siRNA (siCCND1), Cyclin D1 was downregulated, Cyclin D2 (CCND2) was upregulated, and Cyclin D3 (CCND3) expression did not change. Dosing with 50 nM of siCCND2 in addition to 50 nM siCCND1 returned Cyclin D2 levels to a value that was not significantly different from untreated cells. Error bars represent standard deviation (*n* = 3)

Cyclin D1 is one of three Cyclin D homologues, all of which regulate the G1 to S phase transition in the cell cycle.^44^ It has been shown previously that knockdown of Cyclin D1 can cause a compensatory increase in Cyclin D2 expression.^23^ Therefore, we sought to determine if the LNP‐mediated silencing of Cyclin D1 in JeKo‐1 cells provoked similar increases in the expression of homologues Cyclin D2 and Cyclin D3. Figure 3 shows Cyclin D expression following the delivery of siRNA specific against Cyclin D1 at a dose of 50 nM. We observed a decrease in Cyclin D1 (CCND1) levels, a 20‐fold increase in Cyclin D2 (CCND2) levels, and no change in Cyclin D3 (CCND3) levels. This effect was dose‐responsive, with an anti‐Cyclin D1 siRNA dose of 100 nM inducing a 45‐fold increase in Cyclin D2 expression (Supporting Information Figure 3).

This compensatory effect was mitigated by silencing Cyclin D2 in addition to Cyclin D1 (Figure 3). By silencing both of these genes, expression of Cyclin D1 remained low, expression of Cyclin D3 was unchanged, and Cyclin D2 levels were brought back down, close to baseline levels. To control for any delivery vehicle effects in this experiment, the two treated samples each received a total siRNA dose of 100 nM. That is, the first group received a 50 nM siCCND1 dose + 50 nM siControl dose, while the second group received a 50 nM siCCND1 dose + 50 nM siCCND2 dose. It was not clear, however, that silencing Cyclin D2 would be necessary for effective therapy. This is because Cyclin D2 mRNA expression was more than 8,000‐fold lower than Cyclin D1 levels in untreated samples (data not shown).

**Figure 4:**
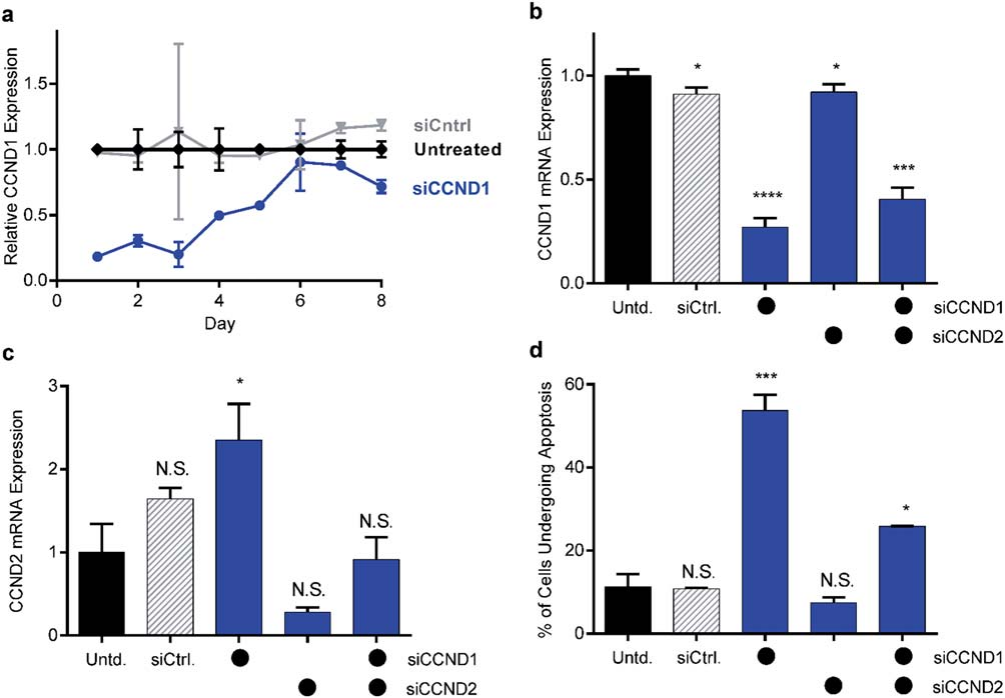
Cyclin D1 silencing increased the rate of apoptosis in JeKo‐1 mantle cell lymphoma cells. (a) A single 50 nM dose of anti‐Cyclin D1 siRNA (siCCND1) suppressed Cyclin D1 mRNA expression for 5–6 days, with maximum silencing of ∼80% occurring 1–3 days post‐transfection. (b) Four days post‐transfection, Cyclin D1 expression decreased following LNP‐mediated delivery of either siCCND1 or a combination of siCCND1 and siCCND2. (c) Delivery of anti‐Cyclin D2 siRNA (siCCND2) silenced Cyclin D2 expression, while delivery of siCCND1 caused Cyclin D2 upregulation. (d) The percentage of cells undergoing apoptosis increased when cells were dosed with siCCND1, but not with siCCND2. In panels (b)–(d), the total siRNA dose for each group was 200 nM, made up of 100 nM doses of a combination of siCCND1, siCCND2, and/or siControl. Error bars represent standard deviation (*n* = 3). Statistically significant differences in gene expression relative to untreated cells were determined using two‐tailed Welch's *t* tests. *, ***, and **** indicate *p* ≤ .05, .001, and .0001, respectively

To determine whether Cyclin D2 siRNA was a necessary inclusion in a therapeutic siRNA cocktail, we measured JeKo‐1 apoptosis rates following Cyclin D1 and Cyclin D2 protein silencing. We first measured the duration of Cyclin D1 mRNA silencing following a single 50 nM dose of siCCND1 to determine appropriate timing for the apoptosis measurement (Figure 4a). Cyclin D1 mRNA levels were silenced by ∼75% for 3 days before returning to baseline by day 6. We, therefore, chose a 4‐day time point for the measurement of gene expression and apoptosis (Figure 4b–d), which should provide enough time for silencing to affect apoptosis. Every sample except the untreated groups received a total siRNA dose of 200 nM to control for any delivery vehicle effects. That is, one group received 100 nM siCCND1 + 100 nM siControl, a second received 100 nM siCCND2 + 100 nM siControl, and a third received 100 nM siCCND1 + 100 nM siCCND2. Together, these data suggest that adding siCCND2 to the siRNA treatment regimen did not improve outcomes. Four days post‐transfection, the “double” Cyclin D siRNA treatment did not result in more apoptosis than when silencing Cyclin D1 alone. In fact, as shown in Figure 4d, apoptosis rates were significantly higher in the siCCND1 group (∼55%) than in the siCCND1 + siCCND2 group (∼25%). Similar results were observed when treating with total siRNA doses of 100 nM (data not shown). Therefore, we decided to target only Cyclin D1 instead of a Cyclin D1‐D2 combination in future experiments.

### 2.3 Multiplexed gene silencing improved apoptosis rates

**Figure 5:**
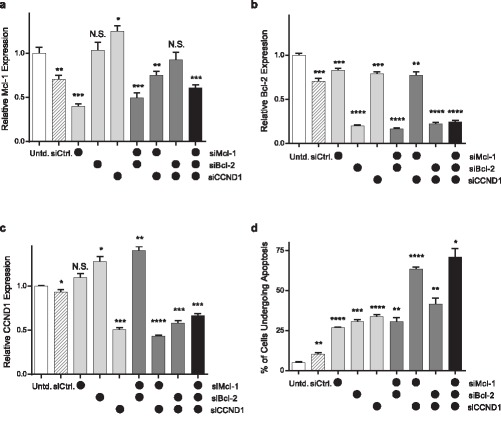
Cocktails of Mcl‐1, Bcl‐2, and Cyclin D1 (CCND1) siRNAs enhanced apoptosis of JeKo‐1 cells compared to single siRNA treatments. JeKo‐1 cells were treated with some combination of siMcl‐1, siBcl‐2, and siCCND1 encapsulated in 306O_13_ LNPs. • indicate LNPs containing 10 nM siMcl‐1 (top row), 100 nM siBcl‐2 (middle row), 100 nM siCCND1 (bottom row) in each graph. Negative control LNPs included control siRNA dosed at 210 nM (representing the maximum siRNA dose in the cocktails). Three days post‐transfection, (a) Mcl‐1, (b) Bcl‐2, (c) CCND1 mRNA expression, and (d) apoptosis rates were determined. Error bars represent SD (*n* = 3). Statistically significant differences in gene expression relative to untreated cells were determined using two‐tailed Welch's *t* tests. *, **, ***, and **** indicate *p* ≤ .05, .01, .001, and .0001, respectively

In previous work, we showed that silencing Mcl‐1 in JeKo‐1 and MAVER‐1 cells using siRNA‐loaded LNPs caused cells to undergo apoptosis.^28^ We thought it may be possible to improve upon these results by targeting multiple genes in one treatment, given the potency of our LNPs. Therefore, we attempted to simultaneously knockdown Mcl‐1, Cyclin D1, and another antiapoptotic protein, Bcl‐2 using a cocktail of the three siRNAs. Figure 5 shows the gene expression and apoptosis resulting from the delivery of seven treatments, which included a triple siRNA cocktail targeting Mcl‐1, Bcl‐2, and Cyclin D1. Every treatment received a total siRNA dose of 210 nM, with siMcl‐1, siBcl‐2, and siCCND1 being dosed at 10, 100, and 100 nM, respectively. For single or double siRNA treatments, the balance dose consisted of control siRNA. Treatment with a 210 nM dose of control siRNA caused a small, but significant, decrease in gene expression and an increase in the fraction of cells undergoing apoptosis. This may be due to loss of viability potentially attributed to the total lipid concentration. We used a lower dose for Mcl‐1 siRNA because we found its ability to cause increased apoptosis maxes out at 10 nM (data not shown).

As can be seen in Figure 5a–c, similar levels of gene silencing occurred whenever the siRNA specific to that gene appeared in the cocktail, regardless of the total number of siRNAs. For example, Bcl‐2 expression was reduced to about 20% of untreated levels whenever siBcl‐2 was included in the siRNA cocktail formulation (Figure 5b). In this experiment, the triple siRNA cocktail resulted in 40, 80, and 35% silencing of Mcl‐1, Bcl‐2, and Cyclin D1, respectively. Interestingly, treatment with siBcl‐2 led to an increase in relative Cyclin D1 mRNA expression (Figure 5c). To our knowledge, this phenomenon has not been reported previously. It is not completely unexpected, however, as these genes are each part of multiple pathways in which feedback mechanisms may occur.^45^, ^46^ Ultimately, siRNA cocktails outperformed single siRNA treatments when considering their effect on apoptosis rates (Figure 5d), with the triple cocktail inducing 75% of JeKo‐1 cells to apoptose 3 days post‐transfection.

Given these positive results, we examined whether or not the formulation procedure for the LNP cocktail affected apoptosis rates. One formulation was made by pre‐mixing the three siRNAs and then formulating the siRNA mixture into LNPs. A second formulation was made by individually formulating each siRNA into their own LNPs and then mixing the three LNP solutions together. Both formulations resulted in comparable levels of JeKo‐1 cell apoptosis (Supporting Information Figure 4), suggesting that the cell entry of LNPs is not an effect‐limiting step in vitro. We recommend the first, pre‐mixed siRNA formulation strategy, as it is simpler.

### 2.4 Multiplexed gene silencing reduced cell proliferation

**Figure 6:**
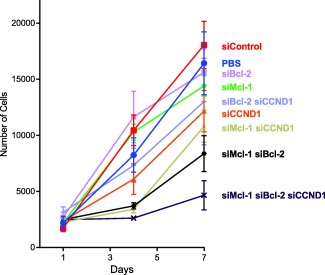
LNP siRNA cocktails targeting Mcl‐1, Bcl‐2, and Cyclin D1 (CCND1) slowed the growth of mantle cell lymphoma cells. Each sample of JeKo‐1 cells received a 200 nM total dose of siRNA encapsulated in 306O_13_ LNPs, with the dose being split evenly between the relevant siRNAs. Saline (PBS) and siControl‐LNP treatments resulted in the highest growth rates, while the triple siRNA cocktail best inhibited cell proliferation. Error bars represent SD (*n* = 3)

Finally, we examined the effect of siRNA cocktails on mantle cell lymphoma growth. In this experiment, JeKo‐1 cells were treated with 200 nM total doses of siRNA in different combinations of siMcl‐1, siBcl‐2, and siCCND1. A combination of LNP solutions that contained all three siRNAs at equal doses nearly completely inhibited cell proliferation 3 days following transfection (Figure 6). While JeKo‐1 cells receiving control LNPs increased in population nine‐fold 7 days after transfection, cells exposed to the triple siRNA cocktail increased only 1.8‐fold. Treatments including only one or two siRNAs against Mcl‐1, Bcl‐2, and/or Cyclin D1 also reduced proliferation to varying degrees compared to control samples.

## 3 DISCUSSION

Mantle cell lymphoma is one of the most deadly subtypes of B‐cell non‐Hodgkin lymphoma.^1^ Although new treatments (e.g., rituximab) have improved outcomes over the last 20 years, survival in the general patient population remains very low.^2^, ^4^ As clinical therapies have progressed from chemotherapy to small molecules drugs to immunotherapy, RNA interference therapy remains an untapped clinical option with the potential to improve treatment outcomes through a unique therapeutic mechanism.

In this study, we formulated LNPs containing synthetic, ionizable, lipid‐like materials, termed “lipidoids,” to deliver siRNA to mantle cell lymphoma cells. Because lipidoid‐containing LNPs had previously been shown to effectively silence genes in a variety of cell types, we sought to examine their ability to transfect notoriously difficult B‐cells. Although several groups have used nanotherapeutics to knockdown Cyclin D1 with siRNA or shRNA in mantle cell lymphoma,^23^, ^24^, ^25^, ^47^ other genes have been largely overlooked.

We found that lipid nanoparticles formulated from the lipidoid 306O_13_ mediate potent and durable silencing in human mantle cell lymphoma cells (Figure 1). We then used these lipidoid nanoparticles to examine the effect of silencing a trio of genes—Cyclin D1, Bcl‐2, and Mcl‐1—on mantle cell lymphoma apoptosis and proliferation rates. The overexpression of Cyclin D1, which occurs in more than 90% of mantle cell lymphoma patients,^5^, ^36^ is correlated with higher rates of tumor cell growth and decreased survival.^48^ We also examined two anti‐apoptotic proteins in the Bcl‐2 family: Bcl‐2, which is commonly overexpressed in mantle cell lymphoma, and Mcl‐1. Mantle cell lymphomas with elevated expression of Mcl‐1 are often associated with high grade morphology and increased proliferation, while benign mantle zone B‐cells are Mcl‐1 negative.^15^, ^16^, ^40^

Targeting Mcl‐1, Bcl‐2, and Cyclin D1 with inhibitors of the respective proteins has generated a good deal of interest due to their aforementioned expression levels and effect on mantle cell lymphoma. Historically, inhibition of the Bcl‐2 family has been shown to have a therapeutic impact on mantle cell lymphoma.^17^, ^19^ However, Bcl‐2 inhibitors and chemotherapeutics use has been stymied by drug resistance due to the upregulation of Mcl‐1.^17^, ^19^ Additionally, Mcl‐1 upregulation has been connected with drug resistance and cancer relapse for a variety of cancers.^49^, ^50^, ^51^, ^52^ Inhibiting Cyclin D1 and Bcl‐2 simultaneously has been shown to improve efficacy in mantle cell lymphoma cells with resistance to Bcl‐2 inhibitors.^45^ Motivated by the therapeutic potential of inhibiting of Mcl‐1, Bcl‐2, and Cyclin D1, but wary of the possible resistance to small molecule drug inhibitors, we chose to target each of the genes at the RNA level using siRNA. As shown in Figure 2, LNPs potently silenced each of these genes in a dose responsive manner.

Knowing that redundancy exists in the Cyclin D pathway, we examined the expression of Cyclin D1, D2, and D3 following Cyclin D1 silencing. Several studies have reported that knocking down Cyclin D1 causes an upregulation of Cyclin D2 in mantle cell lymphoma cells.^23^, ^25^, ^53^ Thus, we wanted to determine if we needed to compensate for a similar upregulation in cells treated with LNPs. As reported in Figures 3 and 4, and Supporting Figure 3, silencing Cyclin D1 in mantle cell lymphoma cells caused an upregulation of Cyclin D2 mRNA expression in a dose and time dependent manner. The highest Cyclin D2 levels were observed in cells treated with highest doses of siRNA at the shortest time points following LNP treatment. Silencing Cyclin D1 had a much larger effect on Cyclin D2 expression than silencing Cyclin D2 had on Cyclin D1 expression (Figures 3 and 4). Klier and colleagues found that Cyclin D1 exists at greater than 1,000‐fold higher levels than Cyclin D2 in JeKo‐1 cells.^53^ Our qPCR analysis also determined that total Cyclin D1 mRNA existed at a much higher level compared to Cyclin D2 mRNA, regardless of treatment (data not shown). This likely explains why the upregulation of Cyclin D2 following the knockdown of Cyclin D1 does not preclude therapeutic effect (i.e., increased apoptosis). Because we found that silencing Cyclin D2 did not increase apoptosis rates (Figure 4d), we chose not to include it as a target in siRNA cocktails.

Multiplexed gene silencing has not previously been attempted for the treatment of mantle cell lymphoma. Simultaneous silencing of multiple gene targets requires a delivery system that is sufficiently potent to induce therapeutic levels of protein knockdown without causing toxicity. Lipid delivery systems, particularly those with a permanent positive charge, can be associated with local toxicity, such as cell irritation and cell lysis, and systemic toxicity causing inflammatory cytokines to be released.^54^

In this study, we demonstrate that a triple siRNA cocktail delivered with nanoparticles made from the ionizable lipidoid 306O_13_ caused increased apoptosis and decreased proliferation in mantle cell lymphoma cells. Based on apoptosis rates (Figure 5), it appears that silencing at least one protein from each pathway (i.e., Cyclin D1 plus either Mcl‐1 or Bcl‐2) results in higher apoptosis rates than when Cyclin D1 is excluded. Figure 6, however, shows greater reductions in proliferation when Mcl‐1 and Bcl‐2 are silenced (compared to Cyclin D1 plus either Mcl‐1 or Bcl‐2). It is possible that the variability associated with mantle cell lymphoma cell culture and LNP formulation and transfection may be responsible for these discrepancies. In both experiments (Figures 5 and 6), the triple cocktail equals or outperforms both two‐component combinations. When taking all of these considerations together, our data suggest that silencing any two of the three target genes is better than silencing any one, and that silencing all three genes is better than silencing any two. Similar multi‐component strategies have been successfully employed to treat several other types of cancer,^55^, ^56^, ^57^ suggesting that LNPs could have broad applicability for cancer treatment.

Further studies are needed to confirm that multiplexed gene silencing kills cancer cells in vivo. Disparate cell populations in vivo may have unique responses to the downregulation of the three genes reported here. Although cancerous cells may die, it is unclear how healthy cells that express one or more of these genes will respond. Some cells may be dependent on one or more of these genes for survival while others are not. Additionally, the expression levels of Cyclin D1, Bcl‐2, and Mcl‐1 genes and proteins will uniquely vary by tissue. To avoid accumulation in healthy cells, either local delivery or the subcutaneous or systemic delivery of actively targeted nanoparticles should be considered. Ultimately, synergy with currently used treatment options, such as chemotherapy, should be examined.

## 4 CONCLUSION

These results highlight the ability of LNPs to deliver a triple siRNA cocktail targeting multiple pathways for therapeutic effect in mantle cell lymphoma. LNPs delivering multiplexed siRNA were more potent than LNPs carrying siRNA targeting a single gene at equivalent siRNA doses. RNAi therapy, which offers a unique mechanism compared to current treatments, has potential to enhance currently available treatment options by increasing their potency while reducing resistance and treatment‐related toxicity.

## 5 MATERIALS AND METHODS

### 5.1 Materials

Tridecyl acrylate (O_13_) was purchased from Pfaltz and Bauer (Waterbury, CT). Cholesterol, *N*,*N*‐dimethyldipropylenetriamine (303), tris[2‐(methylamino)ethyl]amine (304), and 3,3′‐Diamno‐*N*‐methyl‐dipropylamine (306) were purchased from Sigma Aldrich (St. Louis, MO). 1,2 dimyristoyl‐sn‐glycero‐3‐phosophenthanolamine‐*N*‐[methoxy (polyethylene glycol)‐2000] (C14 PEG2000) and 1,2‐distearoyl‐sn‐glycero‐3‐phosphocholine (DSPC) were obtained from Avanti Polar Lipids (Alabaster, AL). Phosphate buffered saline pH 7.4 (PBS), Penicillin Streptomycin, TaqMan Gene Expression Master Mix, TaqMan Gene Expression Assays, High Capacity cDNA Reverse Transcription Kit with RNAse Inhibitor, and Roswell Park Memorial Institute Medium 1640 (RPMI 1640) were purchased from Thermo Fisher Scientific (Waltham, MA). JeKo‐1 and MAVER‐1 human mantle cell lymphoma cell lines were obtained from ATCC (Manassas, VA). Premium Grade fetal bovine serum (FBS) was purchased from VWR Life Science Seradigm (Radnor, PA). Alexa Fluor 488 Annexin V/Dead Cell Apoptosis Kit was purchased from Molecular Probes (Eugene, OR).

Ambion Silencer GAPDH siRNA (human, rat, mouse) was obtained from Thermo Fisher Scientific. All other siRNA sequences were purchased from Sigma‐Aldrich, with siRNA ID numbers as indicated: Mcl‐1 (SASI_Hs01_00162658), Bcl‐2 (SASI_Hs01_00119087), CCND1 (SASI_Hs01_00213908), CCND2 (SASI_Hs01_00043351), CCND3 (SASI_Hs01_00050186), SOX11 (SASI_Hs01_00143858).

TaqMan gene expression assays were purchased from Thermo Fisher Scientific, with Assay IDs as indicated: B‐actin (Hs01060665_g1 FAM), GAPDH (Hs02786624_g1 VIC), CCND1 (Hs00765553_m1 FAM), CCND2 (Hs00153380_m1 FAM), CCND3 (Hs01017690_g1 FAM), Mcl‐1 (Hs01050896_m1 FAM), and Bcl‐2 (Hs00608023_m1 FAM).

### 5.2 Lipidoid synthesis

Lipidoids were synthesized as described previously.^28^ Briefly, three equivalents of tridecyl acrylate (O_13_) were added to one equivalent of N,N‐dimethyldipropylenetriamine (303), tris[2‐(methylamino)ethyl]amine (304), or 3,3′‐Diamno‐N‐methyl‐dipropylamine (306) in a scintillation vial. The mixture was heated and stirred at 90°C for 2 days. The three‐tailed products were isolated using flash chromatography (Isco Teledyne Isco CombiFlash Rf 200 System, Lincoln, NE), with species identities being confirmed by electrospray ionization mass spectrometry.

### 5.3 Lipid nanoparticle formulation

Lipid nanoparticles were formulated as previously described.^28^ Briefly, a lipid solution containing lipidoid, DSPC, cholesterol, and C14 PEG2000 (50:10:38.5:1.5 molar ratio) in ethanol with 5% sodium citrate buffer (pH 3–4) was added to an equal volume of siRNA diluted in sodium citrate buffer. The mixture was briefly vortexed and further diluted 1:1 in PBS (pH 7.4). 306O_13_ LNPs were dialyzed for 4 hr in PBS. siRNA entrapment and particle size were determined using a Quant‐iT Ribogreen RNA Reagent assay (Thermofisher Scientific) and dynamic light scattering, respectively. Particle sizes are reported as number mean.

### 5.4 Cell culture

Immortalized human mantle cell lymphoma cell lines JeKo‐1 and MAVER‐1 were cultured at 37°C in 5% CO_2_ in RPMI 1640 with 20% and 10% FBS, respectively. About 100 U/ml Penicillin Streptomycin was added to the cell culture media.

### 5.5 Gene silencing experiments

Cells were seeded at 250,000 cells/ml in 12 or 24 well plates. LNPs were added such that they made up 10% of the final volume. At denoted time points in each experiment, RNA was extracted using an RNeasy Mini kit (QIAGEN, Germantown, MD) and a QIAshredder (QIAGEN) kit following the manufacturer's protocol. RNA and cDNA concentration were quantified using a NanoDrop 2000c UV‐Vis Spectrophotometer (Thermo Scientific, Wilmington, DE). RNA was reverse transcribed to cDNA using a High‐Capacity cDNA Reverse Transcription Kit with RNase Inhibitor. cDNA was amplified and quantified via RT‐qPCR on a Viia7 Real‐Time PCR system (Applied Biosystems, Grand Island, NY) using TaqMan Gene Expression Master Mix and TaqMan Gene Expression Assay. In experiments depicted in Figures 3 and 4, control (anti‐FVII) siRNA was also incorporated into nanoparticles in order to keep the total siRNA dose consistent across samples.

### 5.6 Cell viability experiment

JeKo‐1 cells were seeded at 250,000 cells/ml in 96 well plates and treated with LNPs as described above. After 24 hr, cell viability was quantified using a MTT Cell Proliferation Assay Kit (ATCC) and Synergy H1 Hybrid Reader (BioTek Instruments, Winooski, Vermont) following the manufacturer's protocol.

### 5.7 Apoptosis experiment

Cells were seeded at 250,000 cells/ml. At several time points between 1 and 8 days following transfection with siRNA‐loaded LNPs, the fraction of cells undergoing apoptosis was determined using an Alexa Fluor 488 Annexin V/Dead Cell Apoptosis Kit and BD Accuri C6 flow cytometer (BD Biosciences, San Jose, CA) following the manufacturer's protocol. FlowJo software was used for flow cytometry data analysis.

### 5.8 Cell counting experiment

JeKo‐1 cells were seeded at 250,000 cells/ml in 24 well plates. Cells were treated with 200 nM doses of total siRNA. The cell count per 20 μl aliquot was determined using a BD Accuri C6 flow cytometer with a HyperCyt Autosampler (Intellicyt, Albuquerque, NM) for high throughput processing. FlowJo software was used for flow cytometry data analysis.

### 5.9 Statistical analysis

All statistical analysis was performed using GraphPad Prism (La Jolla, CA) software. Error bars represent standard deviation (*n* = 3).

## CONFLICT OF INTEREST

The authors have no conflicts of interest to declare.

## Supporting information

Additional Supporting Information may be found online in the supporting information tab for this article.

Supporting Figures
