# Supplementary material for: Lipid nanoparticle siRNA cocktails for the treatment of mantle cell lymphoma
Source: Bioeng Transl Med. 2018 Apr 6;3(2):138–47. doi: 10.1002/btm2.10088 (PMC6063866; doi:10.1002/btm2.10088)
Supplement: Supplementary file 1 — Supporting Figures [file BTM2-3-138-s001.docx]

**Supporting Information**


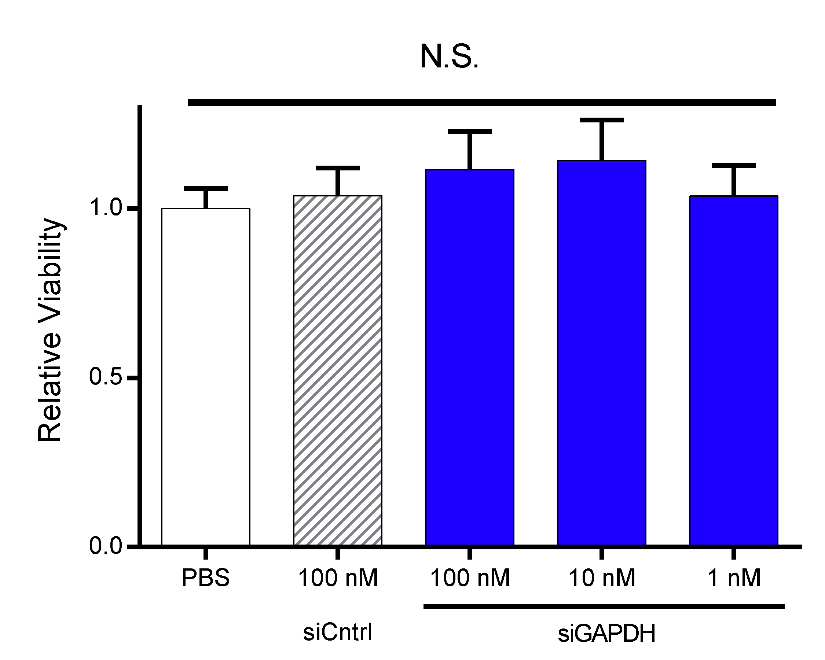


**Supporting Figure 1:** **Treatment with 306O_13_ LNPs caused no reduction in cell viability**. JeKo-1 cells were treated with 1, 10, 100 nM siGAPDH, or 100 nM siGFP (siCntrl in figure) for 24 hours. The cell viability of each treatment group was determined using an MTT assay and compared to untreated cells. Error bars represent standard deviation (n = 3). There was no statistical difference in LNP treated cells compared to untreated cells as determined by a Welch’s t test (p > 0.05).


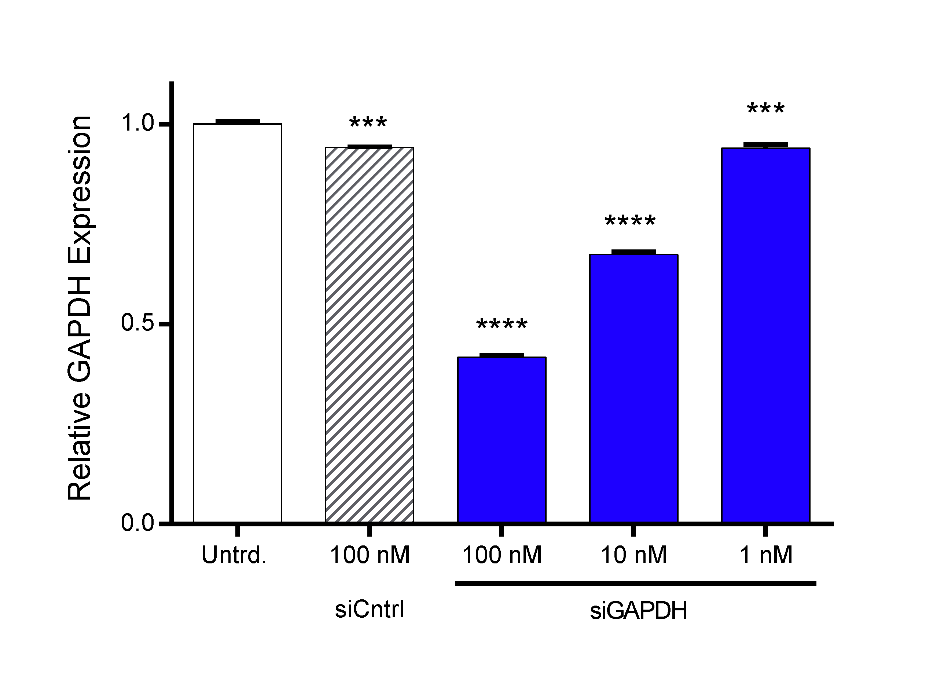


**Supporting Figure 2:** **306O_13_ LNPs silenced genes in the aggressive mantle cell lymphoma cell line, MAVER-1.** Treated cells were dosed with 1, 10, or 100 nM siGAPDH. An LNP negative control was formulated with 100 nM siGFP (siCntrl in figure). Error bars represent standard deviation (n = 3). Statistically significant differences in gene expression relative to untreated cells were determined using two-tailed Welch’s t tests. *** indicates p ≤ 0.001, **** indicates p ≤ 0.0001.


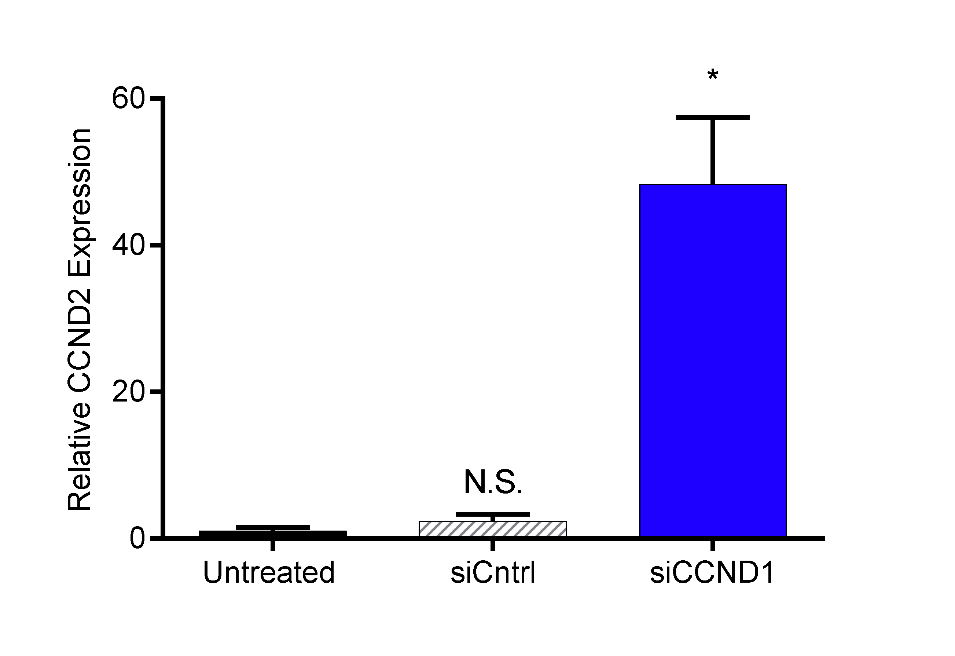


**Supporting Figure 3. Treatment of JeKo-1 cells with siCCND1-loaded LNPs caused a large increase in CCND2 expression 24 hours post-transfection**. JeKo-1 cells were incubated with 100 nM of either siCCND1 or a negative control siRNA (siCntrl) loaded into 306O_13_ LNPs. Error bars represent standard deviation (n = 3). Statistical significance was determined using a Welch’s t test relative to untreated cells. N.S. indicates not significantly different (p > 0.05), * indicates p ≤ 0.05.


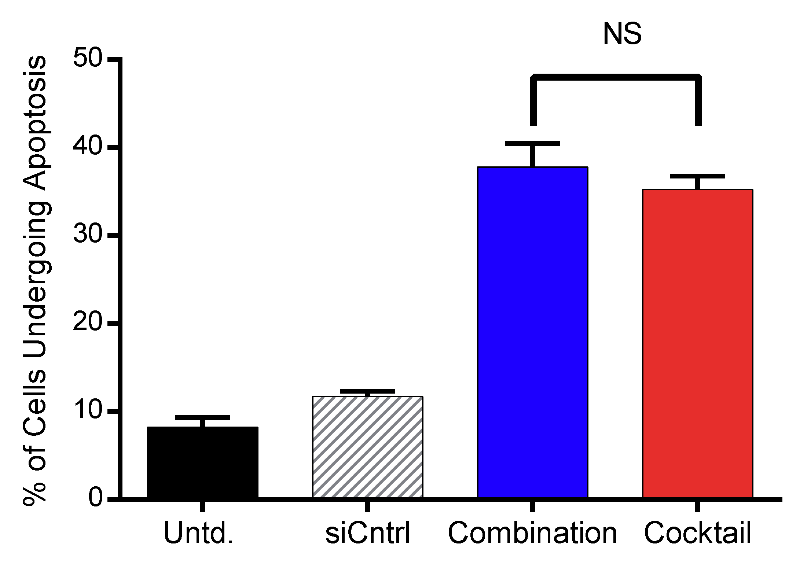


**Supporting Figure 4: LNP cocktail formulation did not affect JeKo-1 apoptosis rates.** JeKo-1 cells were treated with either a “Cocktail” LNP formulated with a pre-mixed solution of anti Mcl-1, Bcl-2, and Cyclin D1 siRNAs or a “Combination” of three LNPs that had each been formulated with a single siRNA. The total siRNA dose for all samples was 300 nM (100 nM of each siRNA). The fraction of cells undergoing apoptosis was determined using an Annexin V/PI assay. Error bars represent s.d. (n = 3). Statistical significance was determined using a Welch’s t test. (N.S. indicates not significantly different (p > 0.05).
